# Molecular Imaging of Galectin-1 Expression as a Biomarker of Papillary Thyroid Cancer by Using Peptide-Functionalized Imaging Probes

**DOI:** 10.3390/biology9030053

**Published:** 2020-03-14

**Authors:** Deborah Fanfone, Dimitri Stanicki, Denis Nonclercq, Marc Port, Luce Vander Elst, Sophie Laurent, Robert N. Muller, Sven Saussez, Carmen Burtea

**Affiliations:** 1 Department of General, Organic and Biomedical Chemistry, UMONS, Avenue Victor Maistriau 19, 7000 Mons, Belgium; Deborah.Fanfone2@alumni.umons.ac.be (D.F.); Luce.Vanderelst@umons.ac.be (L.V.E.); Sophie.Laurent@umons.ac.be (S.L.); Robert.Muller@umons.ac.be (R.N.M.); 2 Center for Microscopy and Molecular Imaging, Rue Adrienne Bolland, 8, 6041 Charleroi, Belgium; dimitri.stanicki@umons.ac.be; 3 Laboratory of Histology, Faculty of Medicine and Pharmacy, University of Mons–UMONS, Avenue du Champ de Mars 6, 7000 Mons, Belgium; Denis.Nonclercq@umons.ac.be; 4 Laboratoire de Génomique, Bioinformatique et Chimie Moléculaire (EA 7528), Equipe Chimie Moléculaire, Conservatoire National des Arts et Métiers (CNAM), HESAM Université, 75003 Paris, France; Marc.Port@lecnam.net; 5 Laboratory of Human Anatomy and Experimental Oncology, UMONS, Avenue du Champ de Mars, 6, 7000 Mons, Belgium; Sven.Saussez@umons.ac.be

**Keywords:** thyroid cancer, galectin-1, peptides, functionalized imaging probes, ultra-small superparamagnetic particles of iron oxide, CF770, magnetic resonance imaging, fluorescence lifetime imaging

## Abstract

Thyroid cancers are the most frequent endocrine cancers and their incidence is increasing worldwide. Thyroid nodules occur in over 19–68% of the population, but only 7–15% of them are diagnosed as malignant. Diagnosis relies on a fine needle aspiration biopsy, which is often inconclusive and about 90% of thyroidectomies are performed for benign lesions. Galectin-1 has been proposed as a confident biomarker for the discrimination of malignant from benign nodules. We previously identified by phage display two peptides (P1 and P7) targeting galectin-1, with the goal of developing imaging probes for non-invasive diagnosis of thyroid cancer. The peptides were coupled to ultra-small superparamagnetic particles of iron oxide (USPIO) or to a near-infrared dye (CF770) for non-invasive detection of galectin-1 expression in a mouse model of papillary thyroid cancer (PTC, as the most frequent one) by magnetic resonance imaging and fluorescence lifetime imaging. The imaging probes functionalized with the two peptides presented comparable image enhancement characteristics. However, those coupled to P7 were more favorable, and showed decreased retention by the liver and spleen (known for their galectin-1 expression) and high sensitivity (75%) and specificity (100%) of PTC detection, which confirm the aptitude of this peptide to discriminate human malignant from benign nodules (80% sensitivity, 100% specificity) previously observed by immunohistochemistry.

## 1. Introduction

As the most common endocrine malignancy, thyroid cancer is increasingly diagnosed worldwide with an incidence rate that is growing steadily and a prevalence that is higher in women [1,2,3,4,5]. According to the American Cancer Society, about 52,890 new cases of thyroid cancer are estimated to occur in 2020, with a clear predominance in women (40,170 women vs. 12,720 men), and about 2180 deaths are caused by this pathology (1140 women vs. 1040 men).

Well-differentiated papillary carcinoma (PC) and follicular carcinoma (FC), as well as anaplastic carcinoma (AC) all originate from thyroid follicular cells. The poorly differentiated AC can arise de novo or from PC or FC. PC accounts for about 70% of thyroid cancers, while FC is less frequent, with an incidence of about 15–20%. Hürthle cell carcinoma is a differentiated thyroid cancer that is less frequent (about 10%) and characterized by a reduced survival rate [5]. Medullary thyroid carcinoma (MTC) is even less frequent, with an incidence rate between 3% and 5% [3,5]. Overall, thyroid cancer mortality is as low as 0.5 cases per 100,000 patients [1], but about 30% of the tumors can generate highly malignant AC characterized by a survival time of less than eight months [4].

Over the past 35 years, the incidence of PC has increased eight-fold in the United States, Europe, and Japan [6,7]. South Korea is the country with the largest increase of incidence to date [8]. Only 4% of thyroid cancers develop distant metastases and the rate of recurrence after treatment remains low (between 7% and 14%). Unlike patients with distant metastases, the five-year survival rate remains excellent (98% vs. 55%) [9]. Patients with PC or FC, being in the vast majority (about 70%) diagnosed as soon as the localized thyroid nodules appear [7], have a favorable prognosis in most cases [10].

Thyroid nodules can occur in 19–68% of the population when identified by high-resolution ultrasound (US), but only 7–15% are confirmed as cancerous [11]. Diagnosis of thyroid nodules relies on fine needle aspiration biopsy (FNAB) combined with cytological analysis. However, this technique is often inconclusive and about 85–90% of the thyroidectomies are performed for benign lesions, when these surgeries could be considered as unnecessary [12]. Moreover, in addition to being psychologically traumatic due to cancer diagnosis, surgical intervention could have secondary consequences on the health and life quality of patients by engendering laryngeal nerve injuries, as well as the hypofunction of parathyroid and thyroid glands if not totally ablated [13,14].

Considering that many of the current serum or histological biomarkers overlap both benign and malign lesions, there is an increasing demand for molecular biomarkers able to discriminate a benign thyroid nodule from a thyroid carcinoma [4,5,15,16,17,18,19,20]. Radioiodine (^131^I or ^123^I) imaging (radioactive iodine uptake test) can be helpful in the diagnosis of thyroid cancer, but only in 10–15% of cases, where FNAB is inconclusive. [^18^F] Fluorodeoxyglucose (FDG) positron emission tomography (PET) is efficient in patients with dedifferentiated thyroid carcinomas, which are poorly iodine avid (negative radioiodine scan). ^123^I-meta-iodobenzylguanidine (MIBG) is useful in some MTCs, the same as [^111^In]octreotide which targets somatostatin receptors, but the efficacy of the latter is rather variable, likely due to the heterogeneous distribution of these receptors. Radiolabeled anti-carcinoembryonic antibodies have shown a sensitivity of 75% in MTC, and an improved sensitivity in more aggressive diseases [12].

Saussez et al. [21] pointed out the diagnostic value of galectin-1 (gal-1) as a serum and cytologic biomarker of PC and AC. This biomarker discriminates the malignant nodules from the benign ones with 80% sensitivity and 97% specificity, respectively [22]. It was also observed that the proliferation rate of thyroid papillary carcinoma-1 (TPC-1) cancer cell line and the AC tumor growth in a mouse model was significantly reduced by the knockdown of gal-1 [22], which represents an additional argument for the diagnostic and therapeutic value of this biomarker.

As a β-galactoside-binding protein, gal-1 belongs to the family of galectins, comprising 15 members involved in various inflammatory and malignant pathologies [23]. Depending on its biological function, gal-1 can be presented both in the form of a 14.5 kDa monomer or as a non-covalent homodimer. Intracellular gal-1 occurs primarily in the form of a monomer and regulates cell growth through protein interactions with cytoplasmic Ras, while extracellular dimeric gal-1 mainly has lectin activity [23].

Several studies have noticed a high rate of gal-1 expression in various cancer cells compared to healthy cells. Many epithelial tumors overexpress this biomarker, including thyroid, colon, and breast cancer, where gal-1 is correlated with tumor aggressiveness and the acquisition of a metastatic profile [23,24]. This protein is involved in the adhesion to the extracellular matrix of cells in hepatocellular carcinoma, melanoma, ovarian, and prostate cancer [25]. Moreover, the secretion of gal-1 is increased during hypoxia and its expression could be used for the prognosis of patients with head and neck cancer [26].

Overall, the literature agrees that gal-1 is overexpressed in cancerous tissues or secreted in the extracellular space of tumor cells and is often related to an aggressive and therefore poor prognosis [27]. In this context, gal-1 exerts two types of functions that are T-cell-related (apoptosis, cell cycle regulation, immunoregulation, tumor evasion of the immune system) or not (cell adhesion, B cell development, mRNA splicing, angiogenesis and differentiation) [23].

Aiming to develop a non-invasive diagnostic strategy able to discriminate benign from malignant thyroid lesions, we previously identified by phage display two gal-1-targeted 12-mer linear peptides, namely P1 and P7 [28], which could be used to functionalize imaging probes for molecular imaging of thyroid nodules and tumors. Their specific binding to gal-1 was confirmed by various methods before and after coupling to ultra-small superparamagnetic particles of iron oxide (USPIO), a contrast agent for magnetic resonance imaging (MRI). The two peptides discriminate thyroid carcinoma from benign thyroid cases with a sensitivity of 65% (P1) and 80% (P7), and a specificity of 67% (P1) and 100% (P7), respectively. After coupling to USPIO, their K_d_ values for gal-1 were comparable and in the order of nanomolar (USPIO-P1: 2.05 × 10^−7^ M; USPIO-P7: 7.56 × 10^−8^ M) due to avidity associated with multivalent presentation on USPIO nanoparticles [28].

The results obtained so far encouraged us to evaluate in the present work the efficacy of both peptides to bind and detect gal-1 in vivo in a mouse model of PC; the binding was observed by two imaging methods, namely MRI and fluorescence lifetime imaging (FLI). For FLI, P1 and P7 were coupled to the near-infrared (NIR) dye CF770.

Among the two imaging methods, MRI has the advantage of being non-invasive and non-irradiating. It offers three-dimensional images of soft tissues, including tumors, of very high resolution. Commonly used in hospitals and preclinical studies in mice, for instance, MRI is based on the magnetic properties of water protons present in different tissues. MRI has excellent anatomical resolution (10–100 µm) and soft tissue contrast [29]. It can be used to visualize the size, location of tumors, and presence of metastases. However, the contrast generated by the intrinsic properties of tissues and the parameters of MRI sequences is often not sufficient to distinguish tissues affected by a pathology from the heathy ones. To help clinicians make a diagnosis, contrast agents are used to enhance this contrast in an area of interest by playing on the T_1_ and T_2_ relaxation times of water protons. Therefore, the contrast of an MRI image can be improved by accumulating the contrast agent in a targeted area. To achieve this goal, these agents are vectored by using ligands targeting specific cellular biomarkers [30].

Optical imaging methods are already employed for clinical diagnosis and many fluorescence-guided surgery procedures are currently under clinical trial [31,32]. This non-ionizing imaging technique provides to the surgeons, in a real-time manner and at a high signal-to-noise ratio (SNR), sensitive molecular information that optimizes tumor resection and thus the patient’s health and life quality. For instance, conventional endoscopy has been coupled with fluorescent imaging probes, increasing the early diagnosis of colorectal cancers [33]. To the best of our knowledge, no optical imaging procedure has yet been proposed for thyroid cancer, although its anatomical accessibility would qualify this pathology as an ideal candidate.

## 2. Materials and Methods

### 2.1. Peptide Synthesis and Coupling to Biotin or to Imaging Probes

Peptides 1 (P1: HLWGWLYAPSFQ) and 7 (P7: YSWHIDIVAPRN) and the non-specific peptide (NSP: HSCNKNSCT) were synthesized as biotinylated (for immunohistochemistry studies) or non-biotinylated (for USPIO coupling) derivatives by Eurogentec (Seraing, Belgium). P1 and P7 coupled to the NIR dye CF770 (P1-CF770, P7-CF770) were synthesized by Bio-Synthesis Inc. (Lewisville, TX, USA). All synthesized peptides have at their N-terminal end a linker of polyethylene glycol (PEG) composed of 2 (for biotin and USPIO derivatives) or 5 (for CF770 derivatives) units of ethylene glycol intended to space them from the chemical moieties dedicated to their detection, here biotin, USPIO, or CF770. This strategy is meant to increase the solubility and accessibility of peptides to the target. The C-terminal end of peptides was blocked by amidation.

**Scheme 1:**
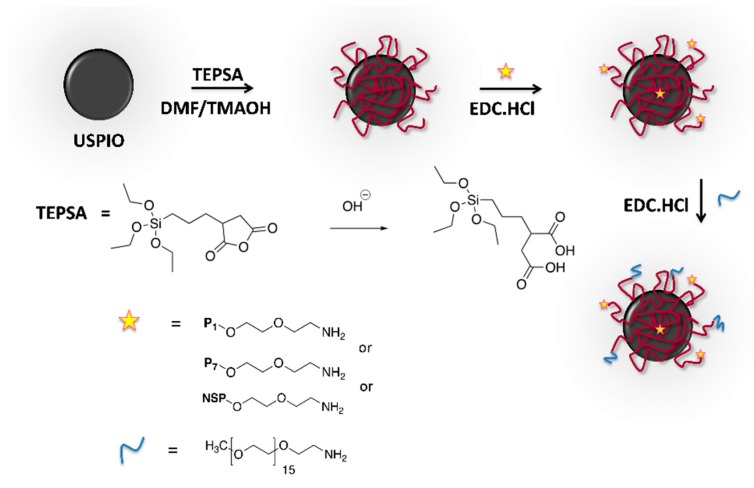
Synthesis of ultra-small superparamagnetic particles of iron oxide (USPIO) derivatives functionalized with peptides P1, P7, or non-specific peptide (NSP).

USPIO coated with an organic layer of polysiloxane (3-(triethoxysilyl)propylsuccinic anhydride, TEPSA, ABCR, Karlsruhe, Germany) exposing carboxylic acid functions were synthesized as previously described [34,35,36]. The peptides were conjugated on the surface of USPIO particles via their *N*-terminal amine groups exposed by PEG following the technique described by Stanicki et al. [34], using *N*-(3-dimethylaminopropyl)-*N*’-ethylcarbodiimide hydrochloride (TCI Chemicals, Tokyo, Japan) as a coupling agent [35,36] (Scheme 1). The chains of O-(2-aminoethyl)-O’-methylpolyethylene glycol (average molecular weight: 750g/mol; Sigma-Aldrich, Overijse, Belgium) were then coupled to saturate the free carboxyl groups and increase the stealth of functionalized nanoparticles. Finally, USPIO derivatives (USPIO-P1, USPIO-P7, USPIO-NSP) were purified by ultrafiltration using a membrane cutoff of 100 kDa (Millipore, Overijse, Belgium). Photon correlation spectroscopy (PCS, Zetasizer NanoS, Malvern Instrument, Worcestershire, UK, equipped with a laser He-Ne working at 633 nm, and a detection angle of 173°) was used to determine the hydrodynamic diameter of USPIO derivatives. For each solution (diluted to 1 mM of iron), 3 measurements (corresponding to Z-average) were recorded over time (time 0, after 2 h, and after 24 h) to check their stability. The Coomassie (Bradford) protein assay kit (Thermo Fisher Scientific, Brussels, Belgium) was employed to determine the number of peptide molecules coupled per USPIO particle, estimated to be of about 4 peptides [37].

### 2.2. Induction and Histological Characterization of the Murine Model of Papillary Thyroid Carcinoma

The Ethical Committee for Animal Welfare of the University of Mons approved the animal experiments performed in this study (ethical permit MU-015-02).

To obtain the mouse model of PC, 5-week-old male Athymic Nude-Foxn1^nu^ mice (Envigo, Venray, The Netherlands) were used. Throughout the induction of PC, the mice were anesthetized by intraperitoneal (i.p.) injection of 50 mg/kg body weight (b.w.) of Nembutal (Sanofi, Brussels, Belgium); simultaneously, an analgesic (buprenorphine; Temgesic^®^, Merck n.v./s.a., Healthcare, Overijse, Belgium, 50 µg/kg) was subcutaneously (s.c.) injected to ensure a mild level of pain.

Before transplantation, the human thyroid papillary carcinoma-1 (TPC-1) cell line was cultured (37 °C, 5% CO_2_) in RPMI 1640 medium with glutamine, supplemented with 10% fetal bovine serum and 1% penicillin/streptomycin (all from Life Technologies, Gent, Belgium).

Various strategies for transplanting TCP-1 cells in athymic nude mice were considered. Using an insulin syringe and a BD Microlance 3 25G (0.5 × 25 mm) needle, 7 × 10^6^ TPC-1 cells/100 µL of complete RPMI culture medium devoid of penicillin and streptomycin were injected s.c. at the thigh level or pseudo-orthotopically (no surgical intervention performed) at the neck level. For neck grafts, the injection was performed through the skin and neck muscles, in the space between the salivary glands, perceiving the thyroid cartilage by palpation and visual observation. The thyroid is located under the thyroid cartilage. The skin was disinfected with ethanol and the cells were then injected at this level. The tumors developed within 2 to 3 weeks without harming the general condition of the animal. Weight and general health were checked regularly. MRI and optical imaging studies were performed when the tumors attained a volume of about 680 ± 460 mm^3^ [28,38].

Nthy-ori 3-1 (normal follicular epithelial cells derived from human thyroid) cell line was also transplanted in mice using the same protocol as for TPC-1 cell line with the aim to develop control tumors presenting a lower gal-1 expression. Before transplantation, Nthy-ori 3-1 cells were grown (37 °C, 5% CO_2_) in RPMI 1640 medium without glutamine, supplemented with 1% glutaMAX, 10% fetal bovine serum, and 1% penicillin/streptomycin (all from Life Technologies). However, these tumors did not develop, as described by specialized literature [39].

At the end of in vivo studies, the mice were euthanized with Nembutal (600 mg/kg b.w., i.p.) and Temgesic^®^ (50 µg/kg, s.c.), and the tumors were sampled, fixed in 4% paraformaldehyde, and paraffin embedded for histological characterization. After dewaxing and rehydration, 5 µm sections were stained using the Masson’s trichrome Accustain^®^ kit (Sigma-Aldrich) before mounting them with Acrytol (Leica Microsystems, Groot Bijgaarden, Belgium). The microphotographs were acquired using a DM2000 Leica microscope equipped with a Leica DFC 425C camera (Leica Microsystems).

### 2.3. Immunostaining of Gal-1 on Healthy Human Organs and Tissues, Cell Models, and Human and Murine PC Tumors

The biopsies of healthy human organs, tissues (kidney, liver, spleen, stomach, duodenum, skeletal muscle), and PC tumors were collected after written informed consent. The histological sections were collected retrospectively and were kindly provided by Dr. Gilles (Anatomopathology Laboratory, EpiCURA, Frameries, Belgium) and by Drs. Salmon and Rorive (Department of Pathology of the Erasme Hospital, ULB, Brussels, Belgium). The ethics committee of the University of Mons approved the study (authorization OM 004).

In addition to TPC-1, gal-1 expression was also assessed in various cells lines (Nthy-ori 3-1, Panc-1 and Capan-2) with the goal of identifying a negative control for in vivo studies. TPC-1 and Nthy-ori 3-1 were cultured as described in Section 2.2. PANC-1 cells (pancreatic carcinoma of ductal cell origin) were cultured in DMEM without pyruvate, supplemented with 10% fetal bovine serum (FBS), 1% non-essential amino acids, and 2% penicillin/streptomycin (all from Life Technologies). Capan-2 cells (human pancreas adenocarcinoma) were grown in Advanced RPMI supplemented with 10% FBS, 1% glutaMAX, and 2% penicillin/streptomycin (all from Life Technologies).

The murine TPC-1 tumors were collected and prepared for histological evaluation as described in Section 2.2.

Gal-1 expression on sections of healthy human organs and tissues and human and murine PC tumors was assessed as previously described [28]. Briefly, sections were consecutively incubated with rabbit anti-gal-1 antibody (Santa Cruz, Heidelberg, Germany), biotinylated goat anti-rabbit antibody, and Vectastain ABC kit (both from Vector Labconsult, Brussels, Belgium). A solution of 0.05% 3,3′-diaminobenzidine (DAB) tetrahydrochloride (Sigma-Aldrich) and 0.02% H_2_O_2_ was used to reveal the gal-1 staining. The counterstaining was performed with Mayer’s hemalum (VWR International, Leuven, Belgium) and the sections were mounted with Acrytol. The specificity of anti-gal-1 antibody used during the immunohistochemistry protocol was validated by ELISA and performed on immobilized human gal-1 (Peprotech, London, UK) or on Protein-Free (Tris-buffered saline, TBS) Blocking Buffer (PFBB, Pierce, Thermo Fisher Scientific). The results revealed perfect recognition of our target but not of the blocking agent PFBB (data not shown in this manuscript).

The biotinylated peptides P1 and P7 bound to sections of healthy human organs and tissues and human PC tumors were detected with a goat anti-biotin antibody, a biotinylated horse anti-goat IgG, and Vectastain ABC Kit (all from Vector Labconsult) as described by Fanfone et al. [28].

To stain gal-1 expressed by various cell lines, the cells were grown in 4-well glass slides (Merck, Overijse, Belgium) [18]. The cells were fixed with 4% formalin (Sigma-Aldrich) and then blocked with streptavidin-biotin blocking kit (Vector Labconsult). After incubating the cells with rabbit anti-gal-1 antibody (Santa Cruz), they were then incubated with horse anti-rabbit antibody conjugated to Dylight 488 and mounted with 4′,6-diamidino-2-phenylindole (DAPI) mounting medium (both from Vector Labconsult) [28].

### 2.4. Studies of USPIO Biodistribution in Mice Bearing TPC-1 Tumors

The biodistribution of USPIO derivatives (USPIO-P1, USPIO-P7, USPIO-NSP) was evaluated by nuclear magnetic resonance (NMR) within various organs and tissues (spleen, liver, kidneys, skeletal muscles) as well as in body fluids (blood, urine) collected from athymic nude mice bearing TPC-1 tumors (*n* = 3/experimental group) induced as described in Section 2.2. The mice were anesthetized (50 mg/kg b.w. of Nembutal, i.p.) and then were injected intravenously (i.v.) in the caudal vein with USPIO derivatives at a dose of 100 µmol Fe/kg b.w. Negative control mice were injected using a similar volume of PBS. The mice were sacrificed (500 mg/kg b.w. i.p. of Nembutal; 4 mg/kg b.w. s.c. of Morphasol analgesic, Dechra, Lille, France) at various intervals after USPIO administration, and organs, tissues, and body fluids were harvested. The blood was collected with heparin by cardiac puncture after opening the rib cage, and plasma was isolated by centrifugation at 7000 rpm for 30 min. The urine was collected by bladder puncture after opening the abdominal wall. The organs and tissues were then collected after transcardial perfusion of PBS to remove the residual USPIO from the cardiovascular system. The biological samples were finally stored at −20 °C before their analysis by NMR.

The uptake of nanoparticles within various organs, tissues, and biological fluids was then analyzed by relaxometry, on a spin analyzer Bruker Minispec mq60 (1.41 T) at 37 °C, at different post-injection times. The transverse relaxation time (T_2_) was therefore measured for all samples. From the T_2_ value, the relaxation rate of water protons, R_2_, was deduced by calculating the reverse of T_2_ (R_2_ = 1/T_2_ (s^−1^)), which is directly proportional to the iron concentration within biological samples. Plasma or urinary concentration of USPIO derivatives was calculated by considering their r_2_ relaxivity values expressed in s^−1^ mM^−1^.

### 2.5. In Vivo Studies by MRI

For MRI studies, athymic nude mice xenografted with TPC-1 tumors were anesthetized with 50 mg/kg b.w. of Nembutal, i.p. Animal breath was monitored throughout the image acquisition using a monitor connected to a sensor placed on the mouse. A warm water circulation system was employed to maintain the body temperature at 37 °C. An internal reference (tube filled with 0.35% agarose) was placed on the side of each mouse to standardize the signal intensity.

The images were acquired using a Bruker Biospec 300 MHz (7T) imaging system (Bruker, Ettlingen, Germany) equipped with a Pharmascan horizontal magnet and a circularly-polarized transmit/receive ^1^H volume coil (internal diameter: 30 mm; 300.3 MHz frequency). After acquiring the pre-contrast images, USPIO derivatives (USPIO-P1: *n* = 7 mice; USPIO-P7: *n* = 5 mice; USPIO-NSP: *n* = 4 mice) were injected i.v. (caudal vein) at a dose of 100 µmole Fe/kg b.w. The images were acquired using a T_2_-weighted rapid acquisition with relaxation enhancement (RARE) MRI sequence with the following parameters: repetition time (TR)/echo time (TE) = 4000/80 ms, RARE factor = 4, number of experiments (NEX) = 4, matrix = 256 × 256 µm, field of view (FOV) = 3.5 × 2.5 cm, slice thickness = 1.1 mm, spatial resolution = 137 × 97 µm, acquisition time (TA) = 17 min 4 s.

ImageJ software (National Institutes of Health, Bethesda, MD, USA) was employed to analyze the tumor images and calculate the contrast enhancement in post-contrast related to pre-contrast images and expressed by the percentage difference in signal-to-noise ratio (ΔSNR%). The signal intensity (SI) was measured within a region of interest (ROI) drawn around the tumor and within the internal reference (IRef), the measurement being reiterated on each image comprising the tumor. The following equation was used to calculate ΔSNR%:

(1) $$\Delta SNR\%=\frac{\left(SI_{post}/IRef\right)-\left(SI_{pre}/IRef\right)\;}{\left(SI_{pre}/IRef\right)}\times 100$$

### 2.6. Staining of USPIO Derivatives in TPC-1 Tumors by Anti-PEG Immuno-Histochemistry

At the end of the MRI studies, the mice were euthanized, and tumors were harvested for histological observation as described in Section 2.2. The binding of USPIO derivatives to the developed tumors was evaluated by immunohistochemistry. The tumor sections were blocked with 0.7% H_2_O_2_ in PBS (15 min) and then with PFBB. They were then successively incubated with 8 µg/mL of anti-PEG antibody made in rat (Abcam, Cambridge, UK; overnight, 4 °C) followed by 30 µg/mL of biotinylated rabbit anti-rat antibody (1 h, room temperature), both diluted in PBS, and then by Vectastain ABC Kit (both from Vector Labconsult). The sections were finally stained with a solution of DAB and 0.02% H_2_O_2_, counterstained with Mayer’s hemalum and mounted with Acrytol. The brown staining was analyzed semi-quantitatively on microphotographs using the ImageJ software as described by Fanfone et al. [28].

### 2.7. In Vivo Studies by Fluorescence Lifetime Imaging (FLI)

The in vivo studies performed by FLI were carried out on 10 athymic nude mice (NU(NCr)-Foxn1^nu^, Charles River Laboratories, L’Arbresle, France) bearing TPC-1 tumors xenografted s.c. at the thigh level. For the acquisition of FLI images, mice were first anesthetized with 4% isoflurane in O_2_ (2 L/min), followed by 2% isoflurane at 0.3 L/min for maintenance.

The images were acquired with a PhotonIMAGER Optima (Ex = 737 nm, Em = 797 nm, TA = 5 s; BioSpace Lab, Nesles la Vallée, France) before and after (1 h, 1.5 h, and 2 h) the i.v. (caudal vein) injection of 800 nmol/kg b.w. of the imaging probes: P1-CF770 (*n* = 3 mice), P7-CF770 (*n* = 3 mice), and CF770 alone (*n* = 4 mice) (VWR International, Leuven, Belgium). At the end of image acquisitions, the mice were euthanized, while tumors and organs (spleen, liver, and kidneys) were harvested for ex vivo biodistribution analysis (lens = 65 mm with f = 2.8, distance to lens = 279 mm).

The fluorescent signal was measured (photons by second, square centimeter, and steradian (ph/s/cm^2^/sr)) on each image by M3Vision software (BioSpace Lab), after drawing regions of interest (ROIs) surrounding the tumors (in vivo and ex vivo) or the organs (ex vivo). The signal was measured on pre- and post-injection images and the signal injected/signal non-injected ratio was calculated. For the ex vivo images, the signal post-injection was normalized to the signal of tissues measured on a non-injected mouse.

### 2.8. Statistical Evaluation

The results are presented as means ± standard deviation (SD). The statistical difference between various experimental groups showing a normal distribution was evaluated by one-way ANOVA (SigmaPlot 11.0 software); a *p*-value <0.05 was considered as significant. The Mann–Whitney test was employed for the data presenting a non-normal distribution.

## 3. Results and Discussion

### 3.1. Gal-1 Expression and Binding of Peptides to Healthy Human Organs and Tissues

In our previous study, we observed higher binding of peptides P1 and P7 in malignant thyroid cases (PC, FC, and AC) than in adenoma or healthy and inflammatory thyroid, this binding being in perfect relationship with gal-1 expression as determined by anti-gal-1 antibody [28]. The immunofluorescent colocalization of peptides P1 and P7, coupled to either biotin or USPIO, with anti-ga1 antibody on TPC-1 cells corroborated their specific interaction with gal-1. Moreover, the ability of USPIO-P1 and USPIO-P7 to displace anti-gal-1 antibody from the binding sites on TPC-1 cells pleads for a specific recognition of gal-1 [28].

**Figure 1:**
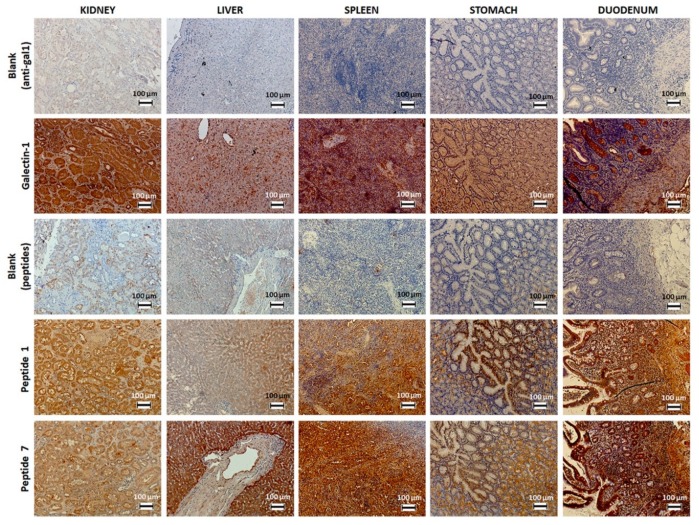
Immunostaining of gal-1 in various human healthy organs using biotinylated peptides 1 and 7 or anti-gal-1 antibody.

Although gal-1 expression and binding of peptides 1 and 7 within various human healthy organs (Figure 1) are not directly informative for thyroid cancer diagnosis, their evaluation could assist in understanding functionalized imaging probes’ biodistribution, subsequent to their interaction with secreted gal-1 or to their uptake by the immune system. Indeed, gal-1 is expressed in all these organs, mainly at the level of the cytoplasm [40], while its presence should not be challenging for thyroid cancer diagnosis due to the distinctive organ location. Moreover, gal-1 could be recognized by the functionalized imaging probes only after its secretion within the extracellular matrix, which is less evident in the case of healthy organs. As opposed to our own results, the Human Protein Atlas reports the absence of gal-1 expression at the protein (but not mRNA) level in organs of the gastrointestinal system including the liver. This dissimilar immunostaining could be explained by the different epitopes identified by polyclonal antibodies on the same protein, generating various levels of immunostaining, but also by the employed immunohistochemistry protocol. Moreover, concerning the studied biopsies, each patient could also present a latent pathology that induces local inflammation and overexpression of gal-1.

In the case of the liver, it has been shown that activated stellate cells overexpress gal-1 in pathological conditions such as liver fibrosis, when the secreted gal-1 stimulates cell growth and proliferation [41]. Accordingly, the liver accumulation of our gal-1-targeted imaging probes could be promoted in pathological conditions associated with gal-1 overexpression and secretion. On the other hand, the uptake by Kupffer cells in the liver and by splenic macrophages represents the main excretion pathway of USPIO derivatives and is responsible for negative signal enhancement of the liver and spleen in MRI with this kind of contrast agent.

The detection of gal-1 within the spleen and other lymphoid organs makes perfect sense. Indeed, several studies have cited the involvement of gal-1 in the regulation of the immune system and in particular during apoptosis of activated T cells via interaction with their membrane receptors (CD7, CD43, CD45). In experimental stress conditions, the serum level of gal-1 and its expression in the spleen and thymus were increased. The colocalization of gal-1 with CD45^+^ lymphocytes in these organs would suggest a role in their apoptosis and thus in the reduction of physiological stress [42].

The presence of gal-1 at the renal level is not new. A previous study already showed its expression in peritubular interstitial tissue and overexpression one week after ischemia [43]. Kidney ischemia is a disease characterized by a significant reduction in glomerular filtration or loss of renal parenchyma. During kidney embryogenesis, mesodermal cells responsible for the development of non-ureteric tubular epithelium express this lectin [44]. It would contribute to the adhesion of these cells to the extracellular matrix and their proliferation [43].

Gal-1 is typically expressed in certain areas of the digestive tract, particularly in the lamina propria [45,46]. A recent study also demonstrated a regulated expression of gal-1 within the inflamed intestinal tissues of patients with celiac disease. The immunoregulatory mechanisms are deregulated in the intestinal mucosa of these patients. Gal-1, found overexpressed in the duodenum of these patients on a gluten-free diet, plays an anti-inflammatory role and allows for their remission [46].

**Figure 2:**
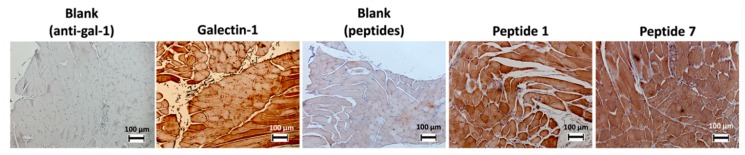
Immunostaining of gal-1 in infrahyoid muscles using biotinylated peptides 1 and 7 or anti-gal-1 antibody.

The expression of gal-1 and the binding of peptides 1 and 7 were also evaluated in the infrahyoid muscles (located near the thyroid gland) in humans. Positive staining of these tissues is observed either by antibodies or by peptides (Figure 2). Several studies found that the presence of gal-1 in smooth and skeletal muscles is related to the level of tissue development. Gal-1 is found in the cytoplasm of the skeletal muscle myoblasts, but when they are merged into a myotube, it is also found in extracellular space. A higher rate of secreted gal-1 was observed during muscle regeneration [47].

### 3.2. Development of the Murine Model of Papillary Thyroid Carcinoma

**Figure 3:**
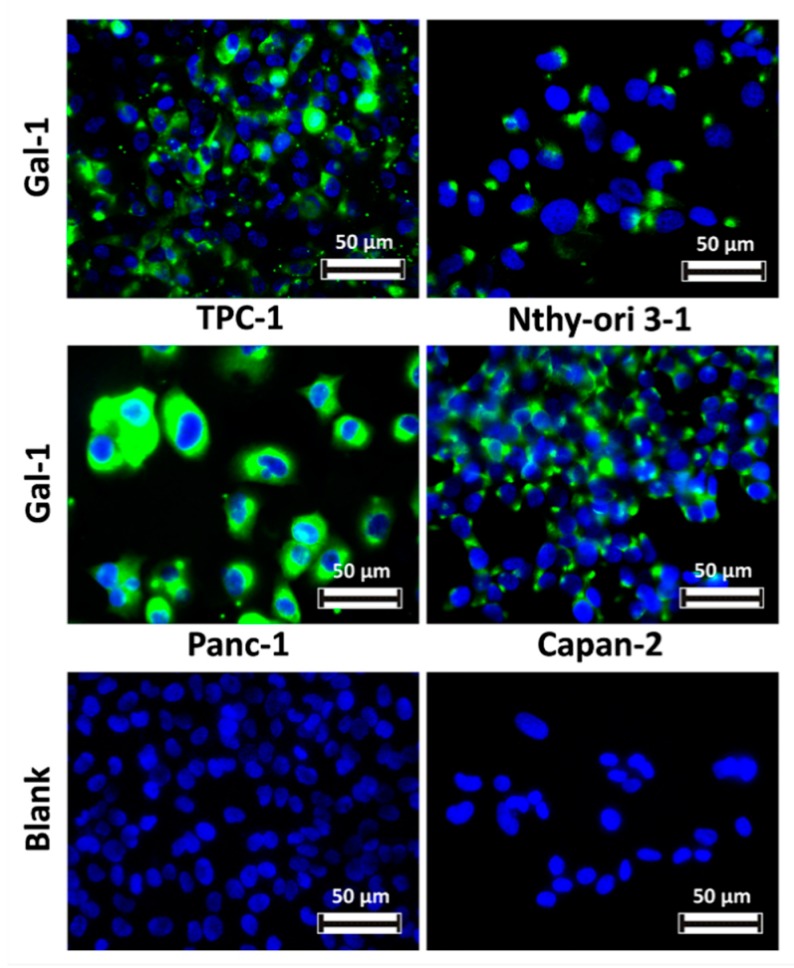
Comparison of gal-1 expression within various human cell lines. Gal-1 was detected with an anti-gal-1 antibody and revealed using the anti-rabbit antibody conjugated to Dylight 488 (green). Nuclei are stained in blue with 4′,6-diamidino-2-phenylindole (DAPI).

An in vivo model of TPC-1 tumors xenografted in immunosuppressed mice has been employed in this work to evaluate the biodistribution of imaging probes, as well as their effectiveness in gal-1 targeting and PC diagnosis by MRI and FLI. In order to identify a cell line that could be grafted in vivo and in this way develop a negative control mouse model (i.e., not expressing or expressing lower levels of gal-1), the expression of the target was evaluated by immunofluorescence on Nthy-ori 3-1, Capan-2, and Panc-1 cell lines; gal-1 expression has been compared to that in TPC-1 cells. By comparing the expression level of our target in human tissues on The Human Protein Atlas, we noticed that the gal-1 protein is not expressed in the pancreas although its mRNA is weakly expressed. For this reason, we decided to verify the expression and the localization of gal-1 in PANC-1 and Capan-2 cell lines. Nevertheless, gal-1 was detected in all studied cell lines at the cytoplasm level (Figure 3), which disqualified them for the generation of a negative control.

Two types of xenografts were considered: an orthotopic one at the neck level and an ectopic subcutaneous one at the thigh level. The absence of trachea obstruction by orthotopic tumors was confirmed by MRI.

The development of a benign tumor model was also attempted by similar injection of Nthy-ori 3-1 cells. Although the target was also expressed by these cells, gal-1 may be confined to the cytoplasm, which could be advantageous for in vivo molecular imaging. Despite the observation by MRI of a small tumor at about five weeks post-transplantation, this was no longer visible one week later either by MRI or by dissection.

A possible negative control model could be represented by gal-1 knockout TPC-1 cells. However, the inhibition of gal-1 expression is not systematically stable over time, as observed by some authors [48], while tumor growth and angiogenesis are halted [22,48]. These technical challenges convinced us to abandon attempts to obtain a negative control animal model.

#### 3.2.1. Morphological Aspects of Papillary Thyroid Carcinoma, Studied by Masson’s Trichrome Staining

**Figure 4:**
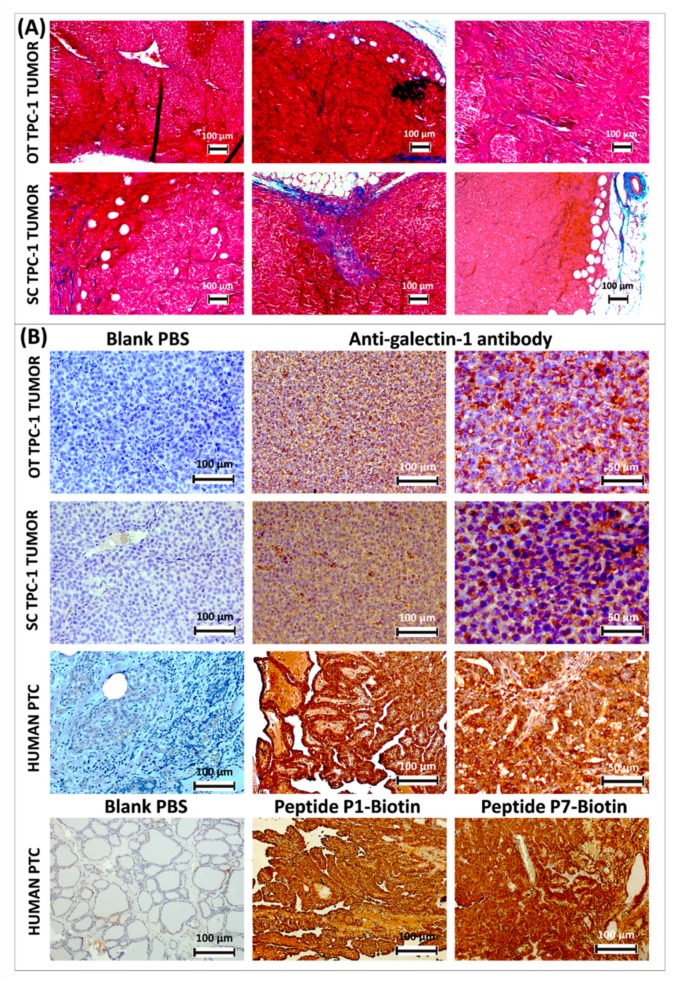
Masson’s trichrome staining of thyroid papillary carcinoma-1 (TPC-1) tumors developed in athymic nude mice orthotopically (OT) at the neck level and subcutaneously (SC) at the thigh level; cytoplasm is stained in red; the collagen is stained in blue (**A**). Immunohistochemical analysis of gal-1 expression (stained in brown) in TPC-1 tumors developed in athymic nude mice and comparison with a case of human papillary thyroid cancer, where gal-1 is immunostained either by anti-gal-1 antibody or by biotinylated peptides P1 and P7; histological sections are counter-stained with Mayer’s hemalum (**B**).

The morphology of orthotopic and ectopic TPC-1 tumors was comparable as observed on histologic sections after Masson’s trichrome staining (Figure 4A). Overall, the presence of collagen fibers stained in blue around the tumors, as well as their invasion within the tumors are noticeable.

Adipocytes are observed at the tumor borders and sometimes within the tumor tissue. According to Zhang and colleagues, stromal cells in white adipose tissue cooperate with the endothelium to promote the formation of blood vessels through the secretion of soluble trophic factors [49]. Several studies suggest that cancer progression would be accelerated in obese patients.

It has long been established that adjacent preexisting vessels represent the sole origin of the neoangiogenic vessels formed within tumors. It appears that the recruitment of progenitor cells, circulating endothelial cells, and several populations of monocytes are also involved in this phenomenon of vasculogenesis [49]. In addition, tumors require the recruitment of mesenchymal stromal cells (MSCs), found in bone marrow and adipose tissue among other tissues. They have the ability to differentiate into various cell types such as osteoblasts, chondrocytes, and adipocytes, and they even generate tumor stromal cells known as cancer-associated fibroblasts. MSCs and endothelial progenitor cells are found in white adipose tissue. They can promote the formation of blood vessels by secreting endothelial growth factor and hepatocyte growth factor among other trophic effects. Cells derived from white adipose tissue can respond to cancerous signals and are then recruited by tumors to promote angiogenesis. Tumor growth was observed as accelerated in obese rodents [49].

Despite the presence of adipose tissue, we did not observe angiogenesis stimulation in our tumor model (Figure 4A,B). It was observed that when cells derived from anaplastic cancer are injected in vivo, they demonstrate a higher tumorigenic potential after orthotopic cell implantation compared to the ectopic subcutaneous transplantation. Immunohistochemical analyses have shown a higher micro-vessel density in the first type of transplant [50]. However, in the case of our papillary tumors, the orthotopic implantation did not seem to stimulate the tumor vascularization.

The limited vascularization in tumors developed from TPC-1 cells can also be explained by comparing them with PC in humans. Vascular invasion occurs in only 2% to 14% of PC cases, more often observed in FC than in PC, where it is thought to be a negative prognostic indicator [51]. Invasion of PC cells occurs instead through lymphatic circulation [52,53]. In a retrospective study of 421 patients with PC, only 9.75% presented a vascular invasion, defined here as a group of tumor cells in close proximity of blood vessels or even projecting within the lumen, in the tumor or around it [51].

Depending on the location of the transplanted TPC-1 cells, it is sometimes possible to observe a fusion of the tumor with glandular or muscle tissue.

#### 3.2.2. Detection of Gal-1 Expression in Murine TPC-1 Tumors

The ectopic subcutaneous and orthotopic TPC-1 tumors developed in nude mice express both the gal-1 detected here by immunohistochemistry and an anti-gal-1 antibody (Figure 4B). If this is secreted, the circulating functionalized imaging probes would have the opportunity to interact with it in vivo. However, gal-1 appears to be heterogeneously expressed and is mostly present in the cytoplasm. It is difficult to attest for its secretion in extracellular space. When comparing with gal-1 expression on sections of human PC, one observes the cytoplasmic staining around the nuclei, especially at the level of the extracellular space. This pattern of immunostaining is observed both with anti-gal-1 antibody and with biotinylated peptides P1 and P7 (Figure 4B).

From a histological point of view, transplanted tumors are characterized by a homogeneous high cell density and the absence of papillae or follicles, as opposed to human PC, where these are characteristic (Figure 4B).

Considering that no significant differences were observed between ectopic subcutaneous and orthotopic TPC-1 tumors xenografted in athymic nude mice both from the viewpoint of morphology and gal-1 expression, we decided to use the ectopic model for subsequent experiments to validate the targeting and diagnostic potential of our imaging probes.

### 3.3. In Vitro and in Vivo Characterization of USPIO Derivatives Functionalized with Gal-1-Targeted Peptides

The choice of the MRI contrast agent to which our gal-1-targeted peptides were grafted was justified by the optimal characteristics of USPIO derivatives. Due to their large iron payload, USPIOs are more sensitive for molecular imaging than paramagnetic contrast agents; they are biocompatible and low-toxic. Our USPIO derivatives (USPIO-P1, USPIO-P7, USPIO-NSP) were first characterized in vitro and later in a mouse model of PC.

#### 3.3.1. Measurement of Hydrodynamic Diameter of USPIO Derivatives and of Their Relaxivity in Biological Fluids

The hydrodynamic diameter of USPIO derivatives was evaluated by PCS. The nanoparticles were diluted to the concentration of 1 mM iron in different solvents (distilled water or PBS). Measurements were taken at 0 time, after 2 h, and after 24 h of incubation to verify their stability (Table 1).

The size of USPIO-P1 particles classifies them as SPIO (diameter larger than 50 nm), while the diameter of USPIO-P7 is close to this limit. The particles functionalized with NSP are then classified as USPIO (diameter less than 50 nm). This difference in size may explain the rapid drop in blood plasma concentration observed in the case of gal-1-targeted contrast agents (please see Section 3.3.2) and thus the significant capture of particles at the liver and spleen level. The less captured USPIO-NSP would have a longer blood plasma clearance allowing it to accumulate in tumors by an enhanced permeability and retention (EPR) effect. Another important point to note is the stability of particles over time, which do not tend to aggregate in PBS over a time interval of 24 h.

The r_1_ and r_2_ relaxivities were measured at 60 MHz and 37 °C on USPIO derivatives diluted in different biological fluids (urine, blood plasma). The USPIO-P1 has higher relaxivity values than the other two derivatives likely due to a higher hydrophobicity of the P1 peptide inducing a binding to the plasma proteins and a subsequent reduction in mobility. Its r_2_/r_1_ ratio in plasma is consequently also higher, moreover related to its larger diameter. This feature anticipates a better potential for negative contrast enhancement by MRI of the targeted sites, but its capture by the reticuloendothelial system (RES) plays against this putative optimal behavior in molecular imaging.

On the other hand, the r_2_/r_1_ values of the other two derivatives correspond to almost half that of USPIO-P1. This characteristic is explained by their higher r_1_ and lower r_2_ values than those of USPIO-P1 in the plasma, conceivably in relationship with their smaller diameter and hydrophilic properties of the P7 and NSP peptides.

#### 3.3.2. Biodistribution of USPIO Derivatives

**Figure 5:**
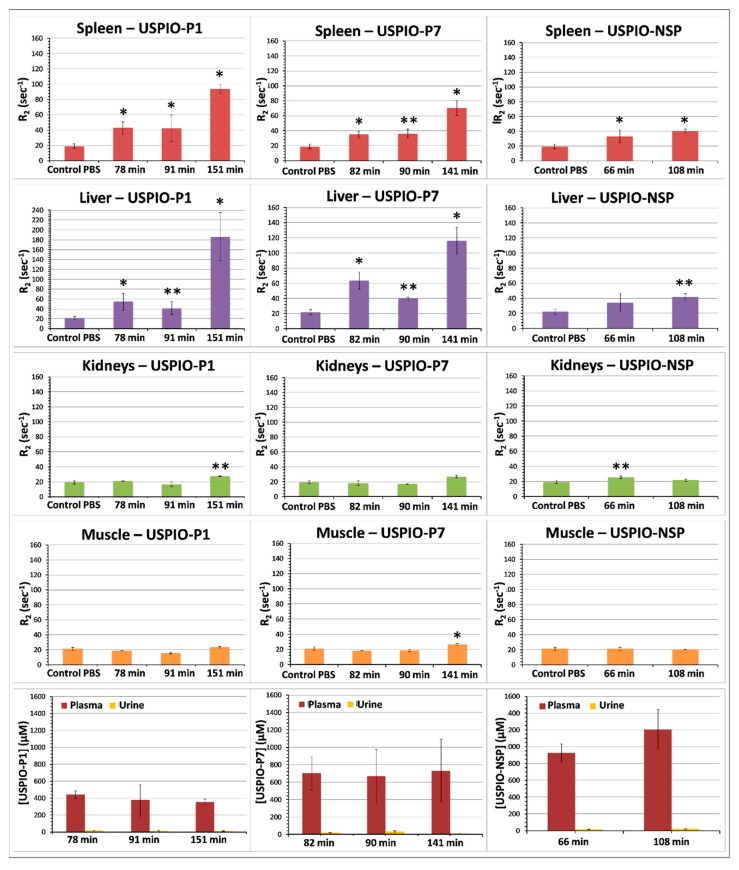
Kinetics of biodistribution of USPIO derivatives determined in athymic nude mice grafted with TPC-1 tumors. Results are expressed as means ± SD. One-way ANOVA was used to evaluate the statistical difference between the test groups and control mice injected with PBS: * = *p* < 0.05; ** = *p* < 0.01.

The biodistribution studies (Figure 5) were conducted after injecting USPIO derivatives into our mouse model of PC, prepared for molecular imaging studies. It is noted that regardless of the post-injection time, the USPIO derivatives did not accumulate in large quantities in the kidneys or muscles, which is an advantage for molecular imaging. Muscles were analyzed because immunohistochemistry studies revealed the binding of anti-gal-1 antibody and of peptides 1 and 7 (Figure 2). Their binding on histological sections is explained by the loss of cellular integrity, which allows the exposure of gal-1. The limited in vivo binding suggests that gal-1 is mainly confined to intracellular space, being inaccessible to interaction with USPIO derivatives.

USPIO derivatives do not appear to be excreted by the kidneys. Accumulation was mainly observed in the spleen and liver for the two gal-1-targeted USPIO derivatives, but especially for USPIO-P1 after more than 2 h post-injection. Uptake by the spleen and liver contributes to the drop of blood plasma concentration of USPIO-P7 and mainly of USPIO-P1 (Figure 5) and reduces the amount of contrast agent available to target gal-1 in tumors. This delayed capture of USPIO derivatives by the spleen and liver suggest that the image acquisitions could be performed before the first 2 h post-injection. The blood plasma concentration of UPSIO-NSP remains high even at ~100 min post-injection, suggesting a slower blood clearance than USPIO-P1 and USPIO-P7. At ~90–100 min post-injection, USPIO-NSP concentration in plasma was around 1000 µM (33.4 ± 7.2% of injected dose, ID), while that of USPIO-P1 and USPIO-P7 was about 400 µM (11.1 ± 4.4% of ID) and 700 µM (22 ± 9.2% of ID), respectively. These results show that more than 50% of ID was cleared from the blood before 1 h post-injection, while the blood concentration of the three USPIO derivatives remained constant between 1–2 h post-injection.

#### 3.3.3. Molecular Imaging of Gal-1 Expression in TPC-1 Tumors by MRI

**Figure 6:**
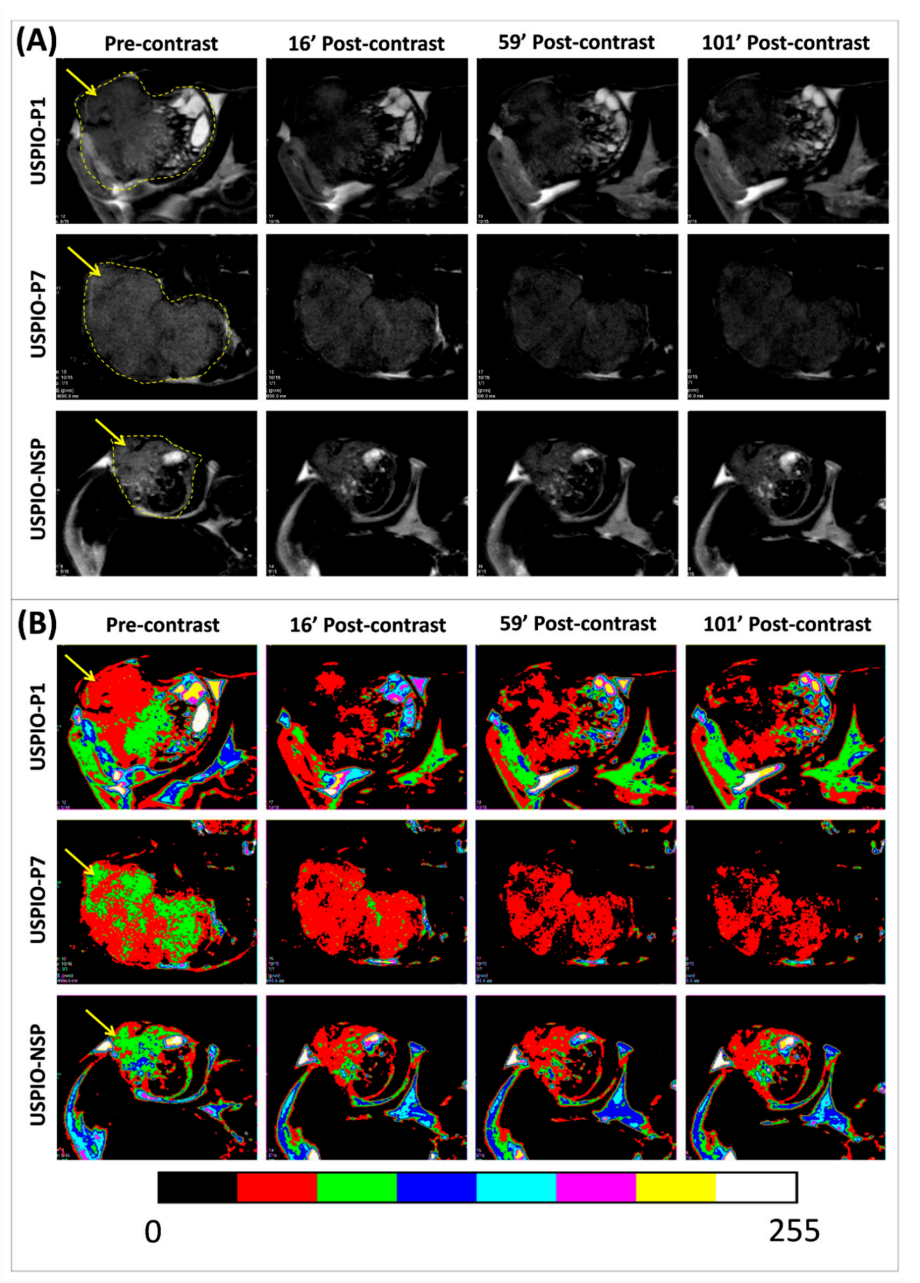
Pre- and post-contrast T_2_-weighted magnetic resonance (MR) images of TPC-1 tumors (pointed by yellow arrows) grafted in athymic nude mice and injected with each one of the USPIO derivatives (**A**). The color masks presented in (**B**) are correlated with the magnitude of negative contrast, observed by the conversion of green or red color in pre-contrast to a red or black color in post-contrast.

T_2_-weighted RARE MRI sequences were employed to follow-up the negative contrast produced by gal-1-targeted USPIO derivatives on TPC-1 tumors xenografted in athymic nude mice (Figure 6). One can observe a slight darkening of tumors after injection of the various USPIO derivatives. USPIO-P7 seems to accumulate in larger quantities and creates a greater negative contrast than the two other USPIO derivatives.

In order to observe the thyroid/pharyngeal area by MRI when TPC-1 tumors are xenografted in orthotopic position and provide the proof of concept of their potential diagnosis, several images were acquired on one mouse in axial and sagittal position at the head and neck level before and after the injection of USPIO-P1 (Figure S1). These images reveal that TPC-1 tumor can be easily distinguished from the surrounding tissues, which do not interfere with the tumor contrast.

**Figure 7:**
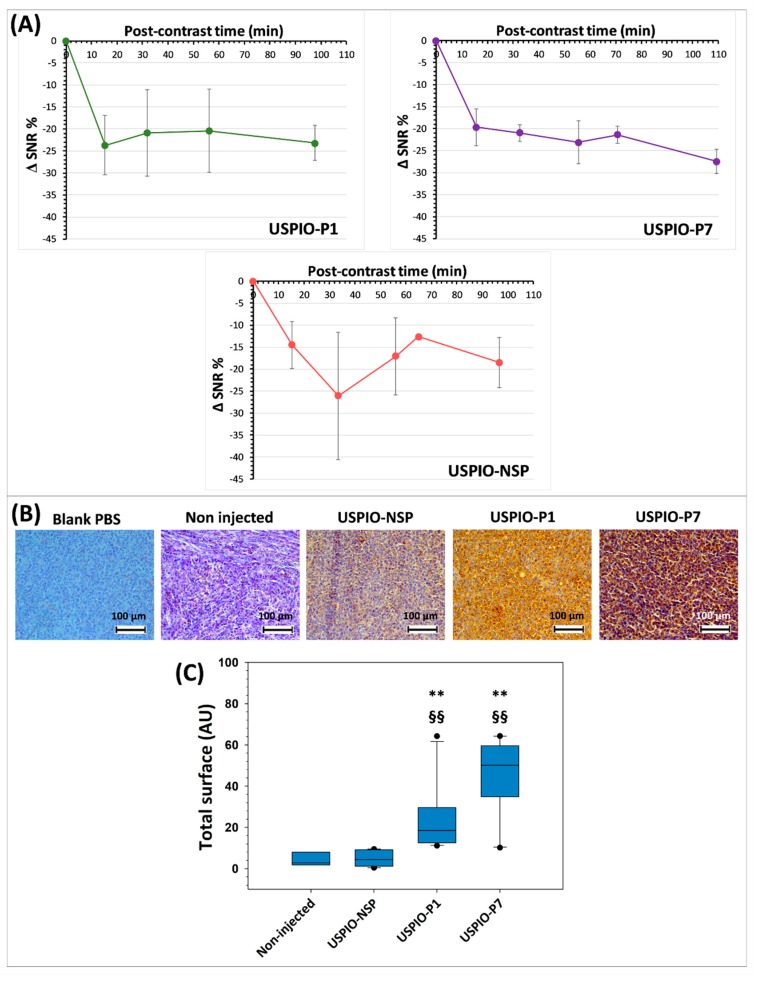
Time evolution of negative contrast (ΔSNR%; signal-to-noise ratio) produced by USPIO derivatives at the level of TPC-1 tumors (**A**). Evidence of the presence of injected USPIO derivatives by anti-polyethylene glycol (PEG) antibody immunostaining in TPC-1 tumors grafted into athymic nude mice (all sacrificed after 130 min post-injection) (**B**). Analysis of the staining of iron nanoparticles by the anti-PEG antibody on TPC-1 tumors (**C**). The results shown in the box and whisker plots are statistically analyzed using the Mann–Whitney test: ** = *p* < 0.01 vs. USPIO-NSP; §§ = *p* < 0.01 vs. non-injected mice.

The evolution of the negative contrast (ΔSNR%) on tumor images until about 100 min post-USPIO injection demonstrates that all three USPIO derivatives produce this effect (Figure 7A). USPIO-P1 and USPIO-P7 appear to have the same targeting efficacy of gal-1 in TPC-1 tumors, although the negative contrast produced by USPIO-P7 is less variable than USPIO-P1. For these two derivatives, there is a maximum decrease of the tumor signal between 20% and 30% after injection.

USPIO-NSP is also present within the tumors as shown by the induced contrast (Figure 7A). The pattern of the contrast curve of USPIO-NSP suggests a variable circulation within the tumor, peaking at around 30 min, which could confirm an EPR capture. Since it is not captured massively by the liver and spleen, we observed that its blood concentration is higher than that of vectorized USPIO, at least during the acquisition of MRI images. This may explain the pronounced EPR effect. USPIO-NSP is therefore not suitable as a negative control for MRI studies of tumors.

There are several reasons for the relatively low contrast observed with gal-1-targeted USPIO derivatives. It may be due to the weak vascularization of TPC-1 tumors, a non-optimal presentation of gal-1-targeted peptides on the surface of USPIO, or the limited secretion of gal-1 within tumors. Indeed, a few small vessels are mainly present at the border of tumors and sometimes within the tumors. If the secretion of gal-1 within tumors is not sufficient, the target’s accession by USPIO could be hampered, which would explain the relatively low contrast observed in MRI. A different tumor mouse model (such as the AC, which is more aggressive) could be considered, in such a way as to observe gal-1 overexpression and eventual secretion. In addition, the circulating concentration of gal-1-targeted USPIO is too low due to the liver and spleen capture (Figure 5). Indeed, our previous research on nanoparticles has shown a plasma concentration greater than 1000 µmol Fe/L at 90–120 min post-injection [54,55,56], whereas during this work, values ranging from about 400 µmol Fe/L (for USPIO-P1) to 700 µmol Fe/L (for USPIO-P7) were found.

Due to the lower capture of USPIO-NSP by the liver and spleen, its blood concentration remains theoretically higher and can therefore accumulate in the tumor.

#### 3.3.4. Detection of USPIO Derivatives in Tumors Developed in Athymic Nude Mice

The presence of USPIO was detected after MRI studies, using an anti-PEG antibody, on the tumors harvested from mice injected with USPIO-P1, USPIO-P7, or USPIO-NSP (Figure 7B). Although USPIO-P1 and USPIO-P7 produce a more important brown staining on tumor sections, nanoparticles are also observed at a lower level on tumors injected with USPIO-NSP. This result corroborates MRI analysis. Indeed, a certain negative contrast of the tumors could be visualized in mice injected with the USPIO-NSP. This could be explained by a nonspecific capture at the tumor level, by an EPR effect.

When the staining produced by the anti-PEG antibody was analyzed and quantified by ImageJ, it was observed that USPIO-NSP labeling is substantially identical to that observed on non-injected mice, while there is a statistical difference (*p* < 0.01) between USPIO-P1 or USPIO-P7 and the two control groups (Figure 7C). The gal-1-targeted USPIO derivatives accumulate at a higher level within the TPC-1 tumors, which is promising. Although not statistically different, the anti-PEG antibody detects more nanoparticles in tumors injected with USPIO-P7 compared to those injected with USPIO-P1, which corroborates the MRI studies.

### 3.4. Molecular Imaging of Gal-1 Expression in TPC-1 Tumors by FLI

There is an urgent need nowadays to develop imaging devices and molecular probes that provide surgeons with real-time feedback aimed to improve the precision of tumor resection. As opposed to the conventional clinical imaging devices, such as MRI, PET, and ultrasound, optical imaging offers potential real benefits for real-time guidance of oncologic surgery by the in situ observation of oncologic molecular targets and in this way improves the therapeutic outcome [31,32]. In this context, our gal-1-targeted peptides coupled to a NIR fluorophore like CF770 (P1-CF770 and P7-CF770) contributed to the discriminative diagnosis of thyroid cancer, its staging, and surgical management.

**Figure 8:**
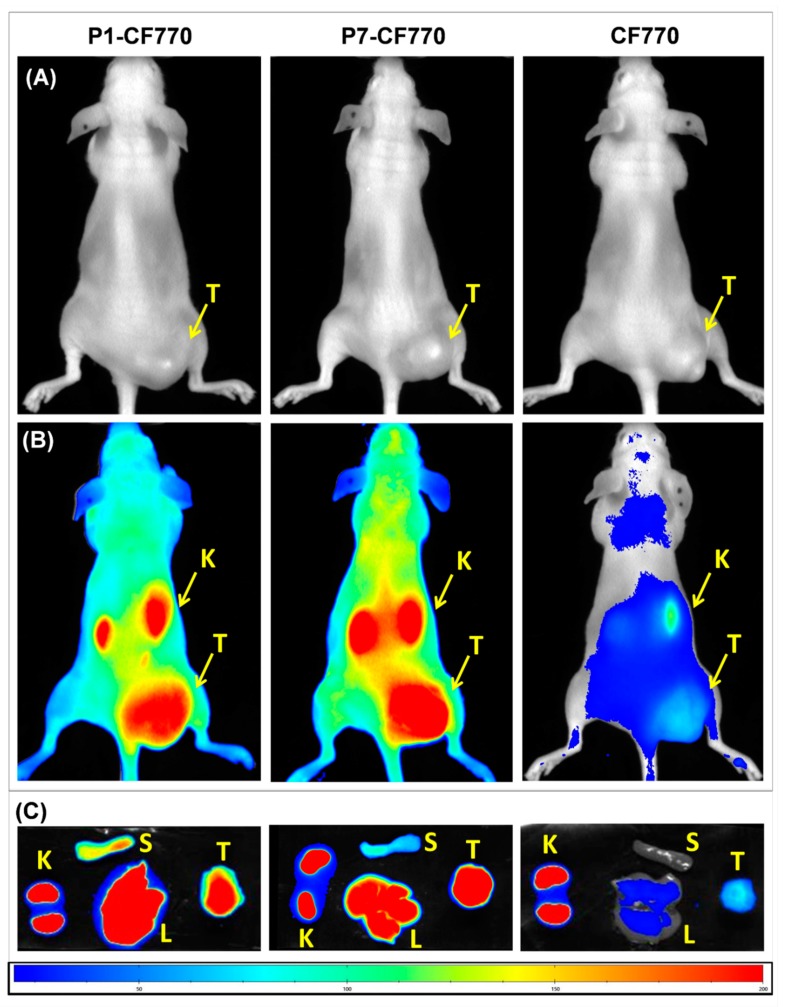
Images acquired by fluorescence lifetime imaging (FLI) of TPC-1 tumors grafted in athymic nude mice before (**A**) and after the injection of P1-CF770, P7-CF770, or CF770 (**B**). Ex vivo FLI images of TPC-1 tumors (**T**), liver (**L**), spleen (**S**), and kidneys (**K**) are shown in (**C**).

The FLI images shown in Figure 8A,B reveal the concentration of gal-1-targeted imaging probes P1-CF770 and P7-CF770 in TPC-1 tumors grafted in athymic nude mice 2 h after administration, while the non-specific imaging probe CF770 is weakly present in tumors, likely as a consequence of the EPR effect. The tumor enhancement observed with P1-CF770 and P7-CF770 is probably explained by the gal-1 binding and is significantly higher than that of the surrounding tissues, where gal-1 is also expressed, in this way allowing a prominent contrast. This tumor retention is furthermore confirmed by ex vivo imaging (Figure 8C), where the difference between the specific and non-specific imaging agents at the tumor level is evident. The three imaging probes are all excreted via the renal pathway. P1-CF770 and P7-CF770 were also found in the liver and spleen, where gal-1 is likewise expressed as explained above.

**Figure 9:**
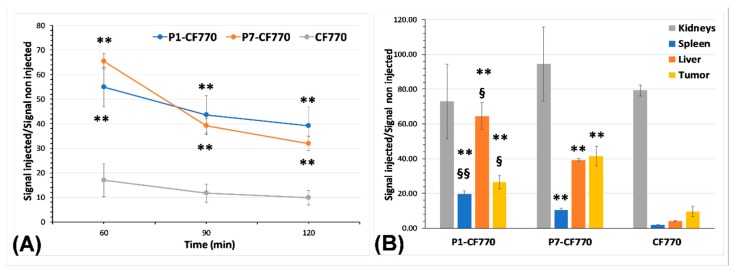
Measurement of the signal intensity using the M3Vision software on in vivo (**A**) and ex vivo (**B**) FLI images. The results are normalized to the signal measured on non-injected mice and organs and are expressed as means ± SD. One-way ANOVA was used to evaluate the statistical difference between the experimental groups: ** = *p* < 0.01 vs. CF770; § = *p* < 0.05, §§ = *p* < 0.01 for P1-CF770 vs. P7-CF770.

The measurement of signal intensity at the tumor level on in vivo images (Figure 9A) highlights the significant (*p* < 0.01) enhancement obtained with P1-CF770 and P7-CF770 as compared to CF770 at the three post-injection times of 60 min, 90 min, and 120 min, respectively. On ex vivo images of tumors and different organs, the signal intensity confirms the significant (*p* < 0.01) tumor uptake of P1-CF770 and P7-CF770 and their uptake in the spleen and liver (Figure 9B), which corroborates with the biodistribution data obtained with USPIO-P1 and USPIO-P7 (Figure 5). The same as the corresponding USPIO derivatives, P1-CF770 is taken at significantly larger levels in the spleen (*p* < 0.01) and liver (*p* < 0.05) when compared to P7-CF770; this last one is significantly (*p* < 0.05) more concentrated in TPC-1 tumors than P1-CF770. The kidney uptake is not significantly different between the three imaging probes.

As explained above, the development of a negative control model of gal-1 expression could not be achieved in mice. Aiming to estimate the sensitivity and specificity of the NIR dye functionalized with gal-1-targeted peptides, we have used the signal measured on the skeletal muscle of the contralateral thigh (contralateral muscle, CLM) as a background level of gal-1 expression (Table 2). The values measured at 2 h after the injection of imaging probes were used for this estimation.

The true positive cases (TP) were defined by the ratio between the signal measured on each tumor (T) and the mean signal + SD of the CLM (T/Mean^CLM^ + SD), being arbitrarily fixed at a value ≥2. The false positive cases (FP) were defined by the ratio between each CLM and the mean signal –SD of T (CLM/Mean^T^ − SD), with this ratio arbitrarily fixed at a value >0.5. For the true negative (TN) cases, the ratio between each CLM and the mean CLM signal (CLM/Mean^CLM^) of each experimental group was considered and set to a value ≤1. Finally, the false negative (FN) cases were defined by the ratio between each T and Mean^T^ (T/Mean^T^) of each experimental group, with this parameter set to <1. These estimations allowed us to calculate a sensitivity and specificity of 50% for P1-CF770 and 25% and 40% for CF770 alone, respectively. On the contrary, P7-CF770 presented a sensitivity of 75% and a specificity of 100%, which corroborates the values estimated for P7 ability to discriminate human thyroid carcinoma from benign thyroid cases determined previously by immunohistochemistry.

## 4. Conclusions

Among well-differentiated thyroid cancers, PC is the most common, accounting for about 70–80% of all thyroid cancers [1,2,3,4,5]. The diagnosis of PC is based on ultrasound assisted FNAB followed by histological analysis of biopsies. However, it turns out that this technique is currently inconclusive in 15–30% of cases [57]. Moreover, about 85–90% of thyroid surgeries are performed for benign nodules, while about 30% imply an inappropriate total thyroidectomy [57,58]. We are thus facing a diagnostic challenge, the distinction between thyroid cancers and benign lesions by a noninvasive diagnostic approach being a prerequisite to prevent unnecessary surgeries, which are traumatic for the patients and expensive for society.

With this in mind, our work was focused on gal-1, a beta-galactoside-binding protein, highlighted as a valuable biomarker for cancer diagnosis, prognostic and treatment. Indeed, gal-1 is overexpressed in a wide range of human cancers (i.e., head and neck, skin, lungs, prostate, ovaries, colorectal region) including thyroid ones, where it is involved in numerous oncological processes, such as immunosuppression, angiogenesis, hypoxia, and metastases [59]. In our previous studies performed on various malignant and benign cases of human thyroid, gal-1 was found overexpressed in AC, PC, and FC, the highest level observed in AC, confirming gal-1 as a biomarker of severe prognosis [28]. According to literature, our gal-1-targeted peptides P1 and P7 are the sole low-molecular weight candidates thus developed to noninvasively discriminate malignant from benign lesions of the human thyroid. Recently, a gal-1-targeted DNA aptamer was proposed for the lung cancer immunotherapy [60].

As opposed to AC, PC is the most frequent thyroid malignancy, but its prognostic is very good. This convinced us to focus our attention on PC diagnosis by proposing two kinds of imaging probes targeted to gal-1. Peptides P1 and P7 were thus coupled either with an MRI probe such as USPIO, or with a NIR probe such as CF770. The efficacy of these functionalized imaging probes to bind and enhance gal-1 expressed in tumors was studied by MRI and FLI in a mouse model of human PC. If optical imaging agents are emerging as promising tools for real-time assistance of oncological surgery [31,32], USPIO derivatives already demonstrated their MRI efficacy and biocompatibility [37,54]. Thanks to their surface coated with a hydrophilic polymer (PEG), their circulation time in the blood is greatly prolonged. In fact, opsonization and degradation by the reticuloendothelial system are reduced, rendering specific tumor targeting more effective.

The expression of gal-1 was observed in human healthy organs, where it is low to medium and the binding of peptides is similar. If gal-1 is secreted within one of these organs, a change in contrast could be obtained at this level. However, it turns out that this protein is secreted globally only in pathological, cancerous, or stressful conditions. The majority of gal-1 is confined to the nucleus and cytoplasm of healthy cells [21,27,61]. It is important to stress that the accumulation of functionalized imaging probes in a given organ is largely explained by the secretion of gal-1, which is directly accessible to these diagnostic compounds. However, when diagnosing thyroid cancer, the area of interest is the thyroid. In other words, when interest is focused on the whole body, if our imaging probes accumulate in a tissue different from the thyroid tumor, it would be wise to further investigate that tissue for possible detection of metastases. On the other hand, gal-1 is constitutively expressed in the mammalian immune system, such as the thymus, lymph nodes, spleen, liver, and bone marrow [62]. This would explain the concentration of gal-1-targeted imaging probes in the spleen and liver, both when P1 and P7 were coupled to USPIO or CF770. This uptake was significantly larger for P1-functionalized imaging probes and could explain the weaker and more variable enhancement of tumors when compared to P7 derivatives. This is furthermore confirmed by the drop in blood plasma concentration of gal-1-targeted USPIO derivatives, which were likely captured by the liver and spleen, both by a specific (i.e., via gal-1 targeting) and a non-specific mechanism (i.e., by phagocytosis due to their larger size), especially in the case of USPIO-P1.

Given the relatively low contrast observed in MRI, it is important to know whether the reason comes from the low-vascularized tumor model or from the design of USPIO derivatives. In fact, the development of imaging probes and the in vivo validation of their diagnostic potential requires further attention. The main challenges to achieve this goal are the optimization of the peptide grafting (i.e., higher number of peptides per particle and optimal exposure to the target) and the stealth of particles. In addition, the accessibility of our imaging probes to the target remains a key point of this diagnostic method. Indeed, the tumor model employed in this work seems to be different from the human PC, both morphologically and from the viewpoint of gal-1 expression, which does not seem to be secreted in large quantities. These different critical aspects therefore require future optimization in order to validate this method of diagnosis.

To validate the effectiveness of gal-1-targeted peptides, another type of probe and therefore imaging of thyroid cancer was explored. Owing to its high sensitivity, in the order of nanomolar, resolution in the range of 50–130 µm, and nonionizing radiation, FLI appears as a promising imaging technique in the field of biomedical research and clinical diagnosis of a wide range of pathologies [63]. In oncology, FLI enables early diagnosis and the depiction of the extent of cancer invasion, improving the accuracy of tumor resection [31]. Our in vivo FLI studies showed that both P1-CF770 and P7-CF770 accumulate in TPC-1 tumors in high concentrations. Considering that P1-CF770 is retained in the liver and spleen at a higher level, its sensitivity and specificity of tumor detection are only 50% in these experimental conditions. The interaction of our imaging probes with cells could be favored by more hydrophobic peptides, such as peptide P1. The hydrophobic character of P1 could promote the non-specific binding of peptide to any cell surface independently of gal-1 expression, although its endocytosis could be higher in cells containing cytosolic gal-1. This may explain the lower sensitivity and specificity observed with P1-functionalized imaging probes. On the other hand, P7-CF770 was able to detect TPC-1 tumors with a high sensitivity (75%) and specificity (100%), which confirms the aptitude of this peptide to distinguish human thyroid carcinoma from benign thyroid cases (sensitivity of 80%, specificity of 100%) observed by immunohistochemistry on FNA biopsy samples previously studied [28]. It is also noticeable that P7 interacts with a higher number of amino acid residues in the CRD core of gal-1, which explains its higher affinity towards gal-1 both before and after coupling to USPIO; it is able to disclose anti-gal-1 antibodies from the binding sites on TPC-1 cells with a stronger efficacy than P1, and induces a more important and constant contrast of TPC-1 tumors when coupled to USPIO or CF770 [28]. With its LogP of 1.02 and LogD_7.4_ of −7.63, P7 is also more hydrophilic than P1 [28], which highlights this peptide as an optimal candidate for the development of gal-1-targeted imaging probes and the diagnosis of malignant thyroid lesions.

## Figures and Tables

**Table 1:** Hydrodynamic diameter values (nm) of vectorized nanoparticles as a function of solvent over time. The relaxivity values r_1_ and r_2_ were measured at 60 MHz and 37 °C.

USPIO Derivatives		Diameter (nm)		r_1_ (s^−1^)		r_2_ (s^−1^)		r_2_/r_1_	
		H_2_O	PBS	Urine	Plasma	Urine	Plasma	Urine	Plasma
	T_0_	67	69						
USPIO-P1	T_2h_	65	67	12.7	12.8	138.2	161	11	12.6
	T_24h_	68	67						
	T_0_	45	44						
USPIO-P7	T_2h_	47	43	8.4	17	84.8	121	10	7.1
	T_24h_	51	41						
	T_0_	26	28						
USPIO-NSP	T_2h_	26	27	8.2	16	81.3	101	10	6.3
	T_24h_	27	27						

**Table 2:** Estimation of true positive (TP), false positive (FP), true negative (TN), and false negative (FN) cases within the experimental groups of mice injected with P1-CF770, P7-CF770 or CF770 alone. The results obtained at 2 h after the injection of imaging probes were normalized to the signal measured on non-injected mice and exploited to finally estimate the sensitivity and specificity of the three near-infrared (NIR) imaging probes.

Parameter	P1-CF770	P7-CF770	CF770
TP T/Mean^CLM^ + SD ≥ 2	1.4	2	2.5
	1.3	2	1.6
	2	2.2	1.1
			0.7
Number of cases	1	3	1
FP CLM/Mean^T^ − SD > 0.5	0.7	0.4	0.9
	0.5	0.5	0.6
	0.8	0.5	0.6
			0.5
Number of cases	2	0	3
TN CLM/Mean^CLM^ ≤ 1	1	0.8	1.4
	0.7	1	0.9
	1.2	1.2	0.9
			0.9
Number of cases	2	2	3
FN T/Mean^T^ < 1	1	0.9	1.5
	0.8	1	1
	1.3	1.1	0.7
			0.9
Number of cases	1	1	2
Sensitivity	50%	75%	25%
Specificity	50%	100%	40%

Note: T = tumor; CLM = contralateral muscle; SD = standard deviation.

## Supplementary Materials

The following are available online at https://www.mdpi.com/2079-7737/9/3/53/s1. Figure S1: Pre- and post-contrast T2-weighted MR images of TPC-1 tumor grafted orthotopically at the neck level in athymic nude mice and injected with USPIO-P1. The images were acquired in axial and sagittal position at the head and neck level. Tu: tumor; Mu: muscle; Th: trachea; SC: spinal cord; Br: brain.
