# Supplementary material for: Molecular Imaging of Galectin-1 Expression as a Biomarker of Papillary Thyroid Cancer by Using Peptide-Functionalized Imaging Probes
Source: Biology (Basel). 2020 Mar 14;9(3):53. doi: 10.3390/biology9030053 (PMC7150867; doi:10.3390/biology9030053)
Supplement: Supplementary file 1 [file biology-09-00053-s001.pdf]

Supplementary material: Figures

Molecular imaging of galectin-1 expression as a biomarker of papillary thyroid cancer by using peptide-functionalized imaging probes

Deborah Fanfone [1†], Dimitri Stanicki [2], Denis Nonclercq [3], Marc Port [4], Luce Vander Elst [1], Sophie Laurent [1,2], Robert N. Muller [1,2], Sven Saussez [5] and Carmen Burtea [1*]

[1] Department of General, Organic and Biomedical Chemistry, UMONS, Avenue Victor Maistriau 19, 7000 Mons, Belgium; Deborah.Fanfone2@alumni.umons.ac.be, Dimitri.Stanicki@umons.ac.be, Luce.Vanderelst@umons.ac.be, Sophie.Laurent@umons.ac.be, Robert.Muller@umons.ac.be, Carmen.Burtea@umons.ac.be

[2] Center for Microscopy and Molecular Imaging, Rue Adrienne Bolland, 8, 6041 Charleroi, Belgium

[3] Laboratory of Histology, Faculty of Medicine and Pharmacy, University of Mons – UMONS, Avenue du Champ de Mars 6, 7000 Mons, Belgium; Denis.Nonclercq@umons.ac.be

[4] Laboratoire de Génomique, Bioinformatique et Chimie Moléculaire (EA 7528), Equipe Chimie Moléculaire, Conservatoire National des Arts et Métiers (CNAM), HESAM Université, 75003 Paris, France; Marc.Port@lecnam.net

[5] Laboratory of Human Anatomy and Experimental Oncology, UMONS, Avenue du Champ de Mars, 6, 7000 Mons, Belgium; Sven.Saussez@umons.ac.be

† Present address: Centre de Recherche en Cancérologie de Lyon, Département Signalisation de l'échappement tumoral. Laboratoire « Cancer cell death », 28, rue Laënnec, 69008 Lyon, Deborah.Fanfone@lyon.unicancer.fr, fanfonedeborah@gmail.com

* Correspondence: Carmen.Burtea@umons.ac.be; Tel.: +32.65.37.38.14

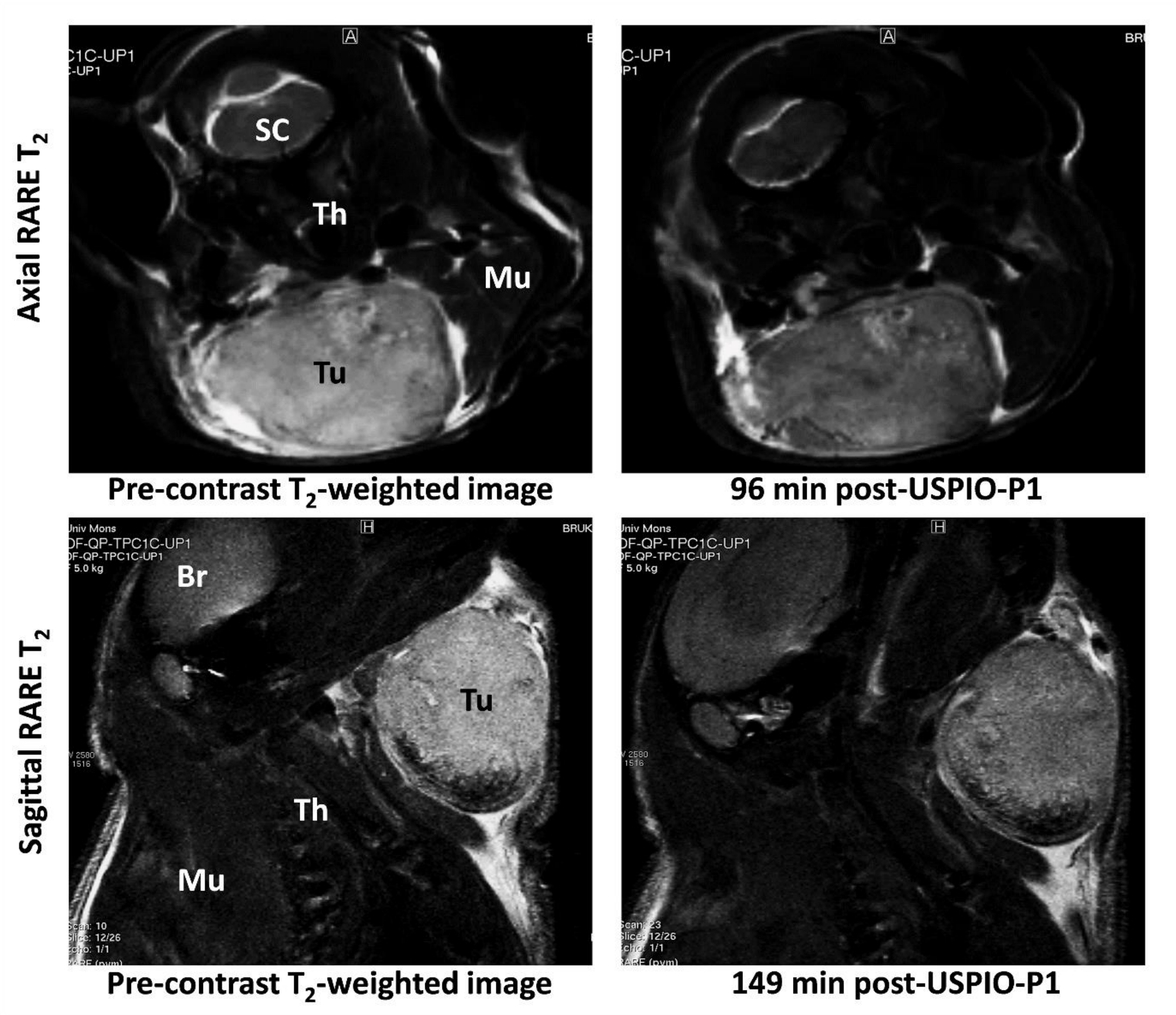

Supplementary Figure S1. Pre- and post-contrast T_2-weighted MR images of TPC-1 tumor grafted orthotopically at the neck level in athymic nude mice and injected with USPIO-P1. The images were acquired in axial and sagittal position at the head and neck level. Tu: tumor; Mu: muscle; Th: trachea; SC: spinal cord; Br: brain.
